# Complete mitochondrial genome and the phylogenetic position of *Mycerobas carnipes* (Passeriformes Fringillidae)

**DOI:** 10.1080/23802359.2021.1909437

**Published:** 2021-04-20

**Authors:** Xiaofeng Zheng, Shangmingyu Zhang, Chuang Zhou, Min Zhu, Yongjie Wu

**Affiliations:** a College of Life Sciences, Key Laboratory of Bioresources and Ecoenvironment, Ministry of Education, Sichuan University, Chengdu, China; b College of Life Sciences, Sichuan Key Laboratory of Conservation Biology on Endangered Wildlife, Sichuan University, Chengdu, China; c The General Work Station of Protected Area of Sichuan Province, Chengdu, China

**Keywords:** *Mycerobas carnipes*, grosbeak, mitochondrial genome, phylogenetic analysis

## Abstract

The complete mitochondrial genome of *Mycerobas carnipes* was sequenced in this study and the total length is 16,806 bp containing 13 protein-coding genes (PCGs), 22 transfer RNA genes (tRNAs), two ribosomal RNA genes (rRNAs), and one control region. The phylogenetic analysis based on 13 PCGs of five grosbeaks and other Fringillidae birds demonstrated that *Mycerobas*, *Coccothraustes*, and *Eophona* had close phylogenetic relationships for clustering as three sister branches, and supported that *Eophona* originated earlier in phylogeny.

*Mycerobas carnipes* is a grosbeak in the family Fringillidae, whose ecological habitats often occur in forest and shrubland and which distributes in northeastern Iran, the Himalayas to the western Ten-zan, central and southwest China (John and Karen 2000). *Mycerobas* and other grosbeak genera are categorized into the subfamily Coccothraustinae removed from the position of tribe Carduelini (Liang et al. 2008). *Mycerobas* is similar in morphology to *Eophona* identified by Clement et al. (1993) and the closely relative relationship between the two genera is further confirmed at the molecular level by Liang et al. (2008) based on CoI gene sequence, by Arnaiz-Villena et al. (2001) and Yang et al. (2006) based on Cytb gene sequence. In addition, *Eophona* is clustered with *Coccothraustes* in the study of Arnaiz-Villena et al. (2007) based on Cytb gene sequence and of Sun et al. (2016) based on 12 protein-coding genes (PCGs) (except ND6 gene), respectively. However, within the grosbeak genera the phylogenetic relationship is ambiguous. According to Zuccon et al. (2012), the topologies of four grosbeak genera (*Coccothraustes*, *Eophona*, *Hesperiphona*, and *Mycerobas*) perform differently using the Bayesian inference (BI), the maximum-likelihood (ML) criteria, and different types of datasets. This study presents the complete mitochondrial genome of *M. carnipes* and constructs a phylogenetic tree based on 13 PCGs of five grosbeaks and other Fringillidae birds for better understanding relationships of grosbeak genera.

The total mitochondrial DNA was extracted from the muscle tissue of *M. carnipes*, which died of airport protection facility for bird strikes in the Ganzi Gesser Airport, Sichuan Province, China (31.75° N, 99.55° E). The specimen was stored in the Natural Museum of Sichuan University with a voucher number of QZKK091. The complete mitochondrial genome of *M. carnipes* was sequenced by Chain Termination Method and the genome sequence has been deposited in the GenBank with the accession MW 304000. The assembly of mitochondrial genome was finished via SeqMan software (version 7.1.0), and the annotation was generated by MITOS first (Bernt et al. 2013), then corrected manually. The sequence of complete mitogenome is 16,806 bp, including 13 PCGs, 22 transfer RNA (tRNA) genes, two ribosomal RNA (rRNA) genes, and one control region. Sequence analysis showed its total base composition as follows: C (31.4%), A (31.2%), T (23.7%), and G (13.6%); the percentage of A + T (54.9%) was higher than G + C (45.1%), similar to other grosbeaks.

The mitochondrial genome sequence of 13 PCGs of *M. carnipes* and other 11 Fringillidae species were used for phylogenetic analysis by BI and ML method (Figure 1). The ML tree was obtained with GTR model with 1000 bootstrap replicates on Mega X (Kumar et al. 2018). A discrete Gamma distribution was used to model evolutionary rate differences among sites (five categories (+G, parameter = 0.3244)). The rate variation model allowed for some sites to be evolutionarily invariable ([+I], 31.18% sites). While the BI tree, constructed by PhyloSuite v1.2.2 (Zhang et al. 2020) with similar partition model determined by ModelFinder (Kalyaanamoorthy et al. 2017) according to AICc, showed completely same topology that the five grosbeaks clustered together sister to *Carpodacus*, *Loxia*, and *Chloris*; and the lineage formed three branches with *Eophona* basal to them. The phylogenetic result supported that *Eophona* had earlier origin than other grosbeaks which was different from previous studies with one or a minority of genes (Arnaiz-Villena et al. 2007; Zuccon et al. 2012). We expect the sequence data will provide a useful data for further study on the phylogenetic evolution of grosbeak genera and other Fringillidae birds.

**Figure 1.**
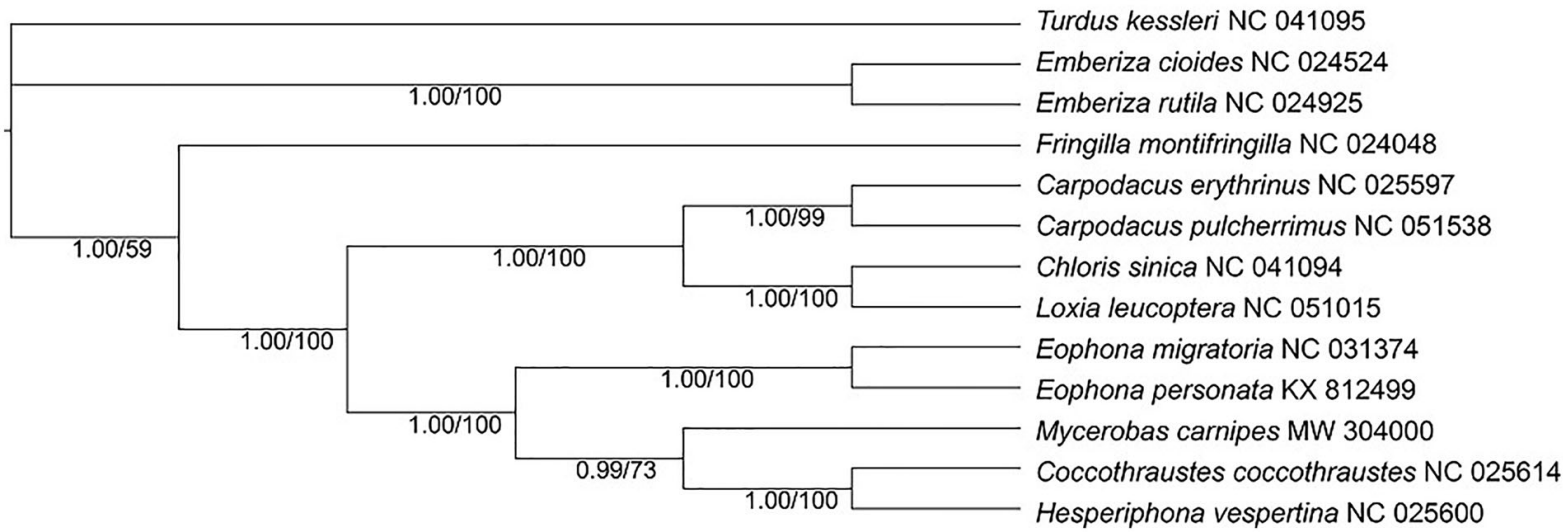
BI tree and ML tree of 12 species from Fringillidae with *Turdus kessleri* as an outgroup, were constructed based on the concatenated dataset of 13 mitochondrial PCGs. The numbers on the node label showed the posterior probabilities and bootstrap values.

## Data Availability

The data are openly available in GenBank of NCBI at https://www.ncbi.nlm.nih.gov, reference number MW 304000.
